# Comprehensive transcriptome mining of the direct conversion of mesodermal cells

**DOI:** 10.1038/s41598-017-10903-z

**Published:** 2017-09-05

**Authors:** Bijan Akbari, Ping Wee, Moein Yaqubi, Abdulshakour Mohammadnia

**Affiliations:** 1 Institute of Biochemistry and Biophysics (IBB), University of Tehran, Tehran, Iran; 2 Department of Medical Genetics and Signal Transduction Research Group, Faculty of Medicine and Dentistry, University of Alberta, Edmonton, AB Canada; 3 Department of Psychiatry, Sackler Program for Epigenetics and Psychobiology at McGill University, Ludmer Centre for Neuroinformatics and Mental Health, Douglas Mental Health University Institute, McGill University, Montreal, Quebec, Canada; 4 Department of Human Genetics, Faculty of Medicine, McGill University, Montreal, Quebec, Canada

## Abstract

The direct reprogramming of somatic cells is a promising approach for regenerative medicine, especially in the production of mesoderm layer-derived cells. Meta-analysis studies provide precise insight into the undergoing processes and help increase the efficiency of reprogramming. Here, using 27 high-throughput expression data sets, we analyzed the direct reprogramming of mesodermal cells in humans and mice. Fibroblast-derived cells showed a common expression pattern of up- and down-regulated genes that were mainly involved in the suppression of the fibroblast-specific gene expression program, and may be used as markers of the initiation of reprogramming. Furthermore, we found a specific gene expression profile for each fibroblast-derived cell studied, and each gene set appeared to play specific functional roles in its cell type, suggesting their use as markers for their mature state. Furthermore, using data from protein-DNA interactions, we identified the main transcription factors (TFs) involved in the conversion process and ranked them based on their importance in their gene regulatory networks. In summary, our meta-analysis approach provides new insights on the direct conversion of mesodermal somatic cells, introduces a list of genes as markers for initiation and maturation, and identifies TFs for which manipulating their expression may increase the efficiency of direct conversion.

## Introduction

The mesoderm is the middle layer of three primary embryonic germ layers, and forms vital organs such as the heart, blood, and bones. Malfunctions to any mesoderm-derived organ bring about serious problems to human health and can lead to the death. For example, it is predicted that cardiovascular disease will be the leading global cause of death, accounting for 23.6 million deaths by 2030^1^. In this regard, providing a solution to treat such abnormalities is a necessary undertaking. The major problem in such disorders is the dysfunction of cells in each organ. Therefore, providing an unlimited source of cells to replace damaged cells is a rational strategy to treat them. The direct conversion of widely available somatic cells to mesoderm-derived cells through the forced expression of transcription factors (TFs) is a promising approach for generating these cells, especially as they do not possess the potential for tumorigenicity posed by the differentiation of pluripotent stem cells^2^.

Fibroblasts are the most common cells of the connective tissue, and are the main cell type used for the direct generation of somatic cells in mice and humans. For example, in previous studies, human fibroblasts have been used to generate osteoblasts^3^, endothelial cells^4–6^, monocytic phagocytes^7^, multilineage blood progenitor (MBP) cells^8^, cardiomyocytes^9–11^, and adipocytes^12^. In addition, fibroblasts have also been used for the direct conversion of somatic cells for mice, for instance, to sertoli-like cells^13^ and hematopoietic progenitor cells^14^. Apart from fibroblasts, the direct reprogramming of other cells to mesoderm layer cells has also been reported. For example, the direct conversion of pre-B cells to macrophages has been reported in three independent studies^15–18^. In addition, Ohno *et al*. showed that PPARg ligands could be used to activate the browning of white adipose tissues^19^. The direct conversion of endothelial progenitors into smooth muscle cells^20^, of T cells to natural killer-like cells^21^, of myoblasts to brown fat cells^22^, and of granulosa cells to sertoli cells^23^, are also among the conversions that have been reported previously. Transcription factors (TFs) are the major driving force behind almost all of the aforementioned conversions. TFs can be used alone or in combination with different TFs to convert various cell types into others. For example, the ectopic expression of POU5F1 alone can reprogram fibroblasts to hematopoietic progenitor cells^8^. The combination of four TFs, SPI1, CEBPA, IRF8 and MNDA, can also convert fibroblasts to monocytic phagocyte^7^.

TFs are the most important factors that direct the progression of many cellular processes, such as development. In this regard, many recent studies have assessed the importance of TFs during cell conversion using *in silico* approaches^24–26^. For example, the analysis of high-throughput genomic expression data sets, including microarrays, RNA-sequencing, and ChIP-sequencing data that corresponds to TF-binding sites can be used to provide a more comprehensive view of the direct conversion process, saving time and costs. Previously, Cahan and colleagues proposed a strategy in which they compared the gene expression profile of wild type *in vivo* cells to their *in vitro* counterparts^24^. The application of such an approach has the capacity to measure the similarity of two cell types in terms of their expression profile and to identify regulators that can be used to generate *in vitro* counterparts with higher efficiency^24^. The most recent computational approach is Mogrify, which is a dedicated platform for identifying the TFs and regulatory networks for the direct conversion of cells^25^. These previous approaches mainly identify the master regulators of conversion. However, in our approach for which we have applied to study the reprogramming of fibroblasts to induced pluripotent stem cells^27^ or the direct conversion of fibroblasts to induced cardiomyocytes^28^, besides identifying these master regulators, we also able to track and highlight the most affected biological processes and reveal common and specific gene expression patterns between generated cells based on their transcriptome profiles. Therefore, our approach allows a deeper level of understanding of the conversion process. Despite extensive efforts in this field, there has not been a comprehensive study to analyze the regulation of the transcriptome during the direct conversion of mesoderm layer cells of humans and mice in order to find the main regulators of the process, as well as the similarities and differences in TFs for these cell conversions.

In this study, we used 27 high-throughput expression data sets to assess the direct conversion of mouse and human mesoderm layer cells for 12 separate types of conversions, with specific attention paid to the direct conversion of human fibroblasts to five different cells: osteoblasts, monocytic phagocytes, endothelial cells, MBPs, and induced cardiomyocytes. To dissect the transcriptome of fibroblast-originated mesodermal cells of humans, we used nine independent data sets and found that during the direct conversion of fibroblasts to the aforementioned five different cells, a group of genes were down-regulated across all generated cells and these genes had common roles in cell cycle regulation and extracellular matrix organization. Furthermore, we found that genes that were up-regulated in all converted cells had a role in the interferon signaling pathway and in the negative regulation of cellular movement. Interestingly, the down-regulation of extracellular matrix genes and up-regulation of negative regulators of cell movement have similar consequences. Therefore, both common up- and down-regulated genes could potentially be used as markers of the initiation of direct reprogramming, as these genes appear to have roles in suppressing fibroblast-specific function. Furthermore, we highlighted genes that were uniquely expressed in a specific converted cell type, and propose that these genes could be considered markers for tracking the termination of the direct conversion process. In addition, for each conversion, we identified the master regulators that most significantly affected the direct conversion process as well as the most affected biological processes. We believe that such a comprehensive approach will give rise to a clearer view of the molecular mechanism beyond the regulation of the gene expression program. In addition, the identification of the master regulators and affected biological processes and their subsequent manipulation may increase the production efficiency of target cells for their use in regenerative medicine. More importantly, highlighting the common and specific genes in mesodermal fibroblast-derived cells provides strong candidate markers for screening the stages of the direct reprogramming process.

## Result

**Figure 1:**
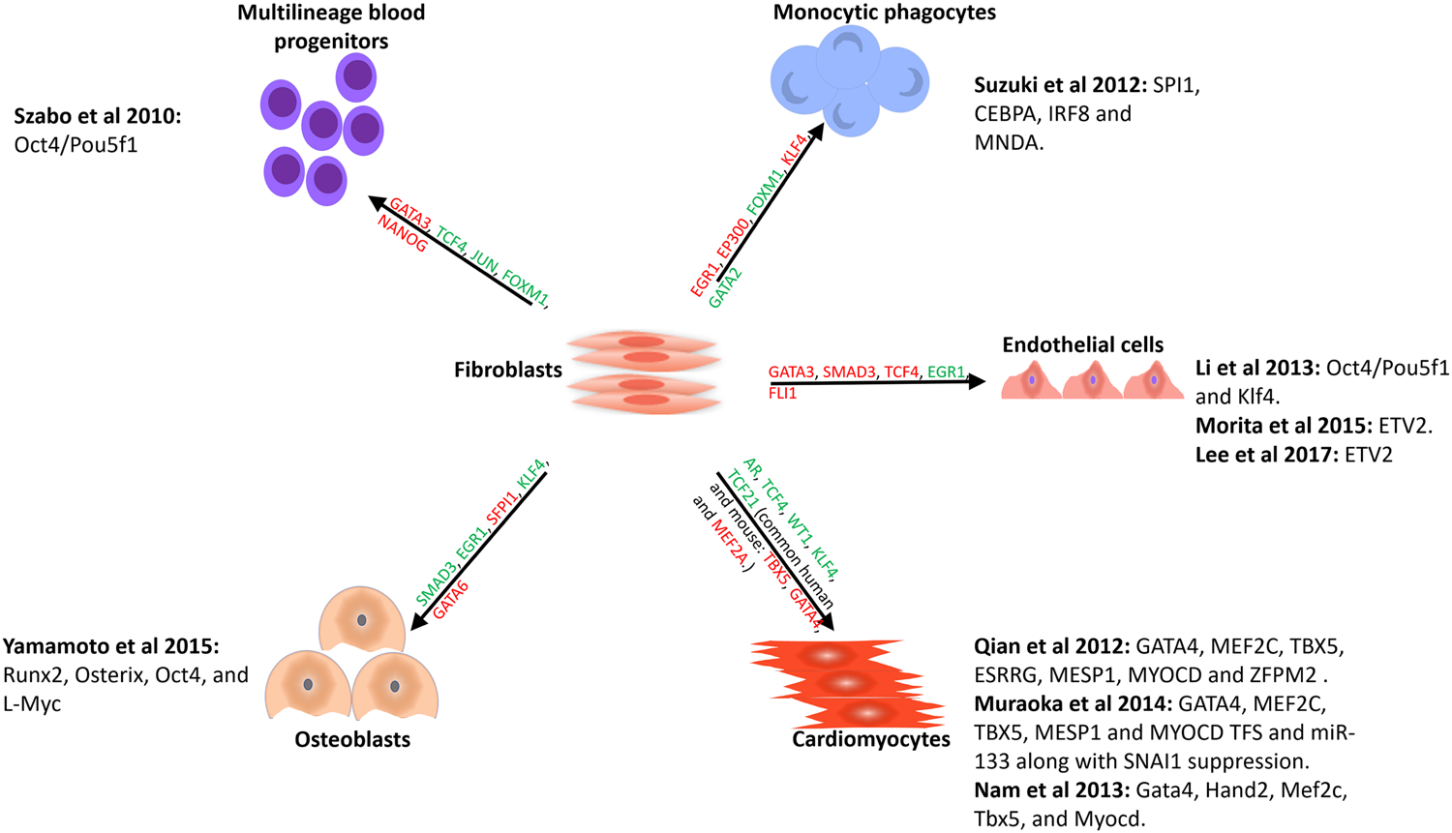
Schematic view of the direct reprogramming of human fibroblasts to five different cells. The top five transcription factors (TF) of each conversion which are identified in our study are represented above arrows. Besides, original TFs which were used to convert cells in the reference studies are indicated next to each cell type. Red and green colors indicate up and down regulation of TFs respectively.

The current study deals with the direct reprogramming of mesodermal layer cells to each other through the meta-analysis of the gene expression profiles of cells obtained from high-throughput transcriptome profiling experiments. In this study, we used 27 publicly available human and mouse data sets corresponding to mesodermal cell direct cell lineage conversions (Table 1). To dissect the underlying mechanisms, we started by analyzing expression data sets, identifying differentially expressed genes (DEGs), acquiring protein-DNA binding sites, constructing the gene regulatory network (GRN), and analyzing the GRN to highlight the main TFs for each conversion. The analysis of the transcriptome of different cell types generated directly from fibroblasts provides the opportunity to identify their similarities and differences, in terms of their commonly regulated genes and processes, along with the cell-specific profiles of each cell type. The results presented in the following sections are broken down into two main parts. In the first part, we considered the direct reprogramming of fibroblasts to osteoblasts, multilineage blood progenitors (MBP), endothelial cells, monocytic phagocytes, and cardiomyocytes in humans (Fig. 1). In the second part, we looked at various direct conversions, including the conversion of amnion cells to induced chondrocyte, of endothelial progenitors to smooth muscle cells, of white adipocyte to brown adipocyte, of T cell to natural killer-like cells, of pre-B cell to macrophage, of myoblasts to brown fat cells, of granolosa to sertoli cell, and of fibroblast to fibroblasts to embryonic sertoli-like cells, paying specific attention to the conversion of pre-B cells to macrophages (Table 1).

**Table 1:** High-throughput expression data sets used for analyzing the direct reprogramming of somatic cells in mesoderm layer.

Study	Conversion	TF	Accession	Organism	Platform
Yamamoto *et al*.^3^	One sample of induced osteoblast (GSM1276644) vs. One sample of fibroblast (GSM1276643)	Runx2, Osterix, Oct4, and L-Myc were used to convert fibroblasts to osteoblasts	GSE52817	Human	Affymetrix Human Gene 1.0 ST Array
Szabo *et al*.^8^	Four samples of Multilineage Blood Progenitor (GSM607078-81) vs. Two sample of fibroblasts (GSM607082-3)	Oct4 was used to convert fibroblasts to multilinegae Blood Progenitor	GSE24621	Human	Affymetrix Human Gene 1.0 ST Array
Suzuki *et al*.^7^	Three samples of induced monocytic phagocytes (GSM675117-9) vs. Three samples of fibroblasts (GSM675105-7)	SPI1, CEBPA, IRF8 and MNDA elements were used to convert fibroblasts to monocytic phagocyte	GSE27304	Human	Illumina HumanWG-6 v3.0 expression beadchip
Qian *et al*.^10^	Three samples of induced cardiomyocytes (GSM1195253-5) vs. Three samples of cardiac fibroblasts (GSM1195229, 1195230-1)	GATA4, MEF2C, TBX5, ESRRG, MESP1, MYOCD and ZFPM2 were used to convert fibroblasts to Cardiomyocytes	GSE49192	Human	Affymetrix Mouse Gene 1.0 ST Array
Muraoka *et al*.^9^	One sample of induced cardiomyocytes (GSM1370498) vs. One samples of fibroblasts (GSM1370494)	GATA4, MEF2C, TBX5, MESP1 and MYOCD TFS and miR-133 along with SNAI1 suppression were used to convert fibroblasts to Cardiomyocytes	GSE56913	Human	3D-Gene Human Oligo chip 25k V2.1
Nam *et al*.^11^	One sample of induced cardiomyocytes (GSM1065980) vs. One samples of fibroblasts (GSM1065982)	Gata4, Hand2, Mef2c, Tbx5, and Myocardin were used to convert fibroblasts to Cardiomyocytes	GSE43588	Human	Affymetrix Human Gene 1.0 ST Array
Li *et al*.^5^	Two samples converted endothelial cells (EC) (GSM1047770-1) vs. One sample fibroblast (GSM1047773)	Oct4 and Klf4 TFs were used to convert fibroblasts to ECs	GSE42672	Human	Affymetrix Human Genome U133 Plus 2.0 Array
Morita *et al*.^6^	Three samples of induced functional endothelial cells (GSM1191056-58) vs. Three samples of skin fibroblasts (GSM1191059-61)	ETV2 TF was used to convert fibroblasts to ECs	GSE48980	Human	Agilent-039494 SurePrint G3 Human GE v2 8 × 60 K Microarray 039381
Lee *et al*.^4^	Two samples of Endothelial cells (GSM1229531-2) vs. Four samples of fibroblasts (GSM1229521-2 and 1229527-8)	ETV2 TF was used to convert fibroblasts to ECs	GSE50801	Human	Affymetrix Human Gene Expression Array
Ishii *et al*.^63^	Six samples of induced chondrocytes (GSM737450-55) vs. One sample of amnion cell (GSM737458)	BCL6, BRACHYURY, c-MYC, MITF, and BAF60C were used to convert amnion cell to chondrocyte	GSE29745	Human	Agilent-014850 Whole Human Genome Microarray 4 × 44 K G4112F
HaYeun Ji *et al*. 2016	Three samples of induced smooth muscle (iSM) cells (GSM1895351-353) vs. Three samples of endothelial progenitor (EP) cells (GSM1895345-7)	Myocardin (MYOCD) element was used to convert EP cells to iSM cells	GSE73469	Human	Affymetrix Human Genome U133A 2.0 Array
Ohno *et al*.^19^	Two samples of brown adipocyte (BA) (GSM860622-3) vs. Two samples of white adipocyte (WA) (GSM860620-1)	Rosiglitazone drug was used to convert WA to BA	GSE35011	Mouse	Affymetrix Mouse Genome 430 A 2.0 Array
Li *et al*.^21^	Four samples of natural killer-like (NK) cell (GSM525091-4) vs. Four samples of T cell (GSM525095-8)	Bcl11b deletion was used to convert T cell to NK cell	GSE21016	Mouse	Illumina MouseWG-6 v2.0 expression beadchip
Bussmann *et al*.^15^	Two samples of macrophage (GSM433189-433204) vs. Two samples of Pre-B cell (GSM433172-3)	C/EBPα TF was used to convert Pre-B cell to macrophage	GSE17316	Mouse	Affymetrix Mouse Genome 430 2.0 Array
Di Tullio *et al*. 2011	Two samples of macrophage (GSM800847-8) vs. two samples of Pre-B cell (GSM800839-40)	C/EBPα TF was used to convert Pre-B cell to macrophage	GSE32330	Mouse	Affymetrix Mouse Genome 430 2.0 Array
Barneda-Zahonero *et al*.^17^	Two samples of macrophage (GSM902526-7) vs. two samples of Pre-B cell (GSM902524-5)	C/EBPα TF was used to convert Pre-B cell to macrophage	GSE36827	Mouse	Affymetrix HT MG-430 PM Array Plate
Kallin *et al*.^18^	Two samples of macrophage (GSM977055-6) vs. two samples of Pre-B cell (GSM977053-4)	Application of C/EBPα TF, along with down regulation of Tet2.	GSE39666	Mouse	Agilent-026655 Whole Mouse Genome Microarray 4 × 44 K v2
Kajimura *et al*.^22^	Three samples of brown fat cells (GSM399083-5) vs, Three samples of myoblasts (GSM399086-8)	PRDM16-C/EBP-beta complex was used to convert Myoblasts to brown fat cell	GSE15895	Mouse	Affymetrix Mouse Genome 430 A 2.0 Array
Uhlenhaut *et al*.^23^	Three samples of Sertoli-like cells (GSM422396-8) vs. Two samples of granulosa cells (GSM422394-5)	Downregulation of Foxl2 TF was used to convert Granulosa cells to sertoli-like cells	GSE16853	Mouse	Affymetrix Mouse Gene 1.0 ST Array
Buganim *et al*.^13^	Four samples of induced embryonic sertoli like cells (ieSC) (GSM973731,973734,973736, 973739) vs. four samples of fibroblasts (GSM973722, 973726, 973732, 973737)	Ectopic expression of Nr5a1, Wt1, Dmrt1, Gata4, Sox9 TFs were used to convert fibroblasts to (ieSC)	GSE35653	Mouse	Agilent-028005 SurePrint G3 Mouse GE 8 × 60 K Microarray
Ieda *et al*.^58^	Three samples of induced cardiomyocytes (GSM554561, 554591-2) vs. three samples of fibroblasts (GSM554761, 554778-9)	Gata4, Mef2c and Tbx5 TFs were used to convert fibroblasts to Cardiomyocytes	GSE22292	Mouse	Affymetrix Mouse Gene 1.0 ST Array
Song *et al*.^59^	One sample of induced cardiomyocyte (GSM909487) vs. one sample of fibroblasts (GSM909483)	GATA4, Hand2, MEF2C and Tbx5 TFs were used to convert fibroblasts to Cardiomyocytes	GSE37057	Mouse	Illumina MouseWG-6 v2.0 expression beadchip
Christoforou *et al*.^60^	Three samples of induced cardiomyocyte (GSM1084562-4) vs. Three samples of fibroblasts (GSM1084553-5)	M2rtTA, GATA4, TBX5, MEF2C, Mesp1, SMARCD3, MYOCD, SRF were used to convert fibroblasts to Cardiomyocytes	GSE44401	Mouse	Affymetrix Mouse Genome 430 A 2.0 Array
Fu *et al*.^61^	Three samples of induced cardiomyocyte (GSM1712854-6 (vs. Three samples of fibroblasts (GSM1712851-3)	Chemical cocktail was used to convert fibroblasts to Cardiomyocytes	GSE69924	Mouse	Affymetrix Mouse Genome 430 2.0 Array
Ifkovits *et al*.^62^	Three samples of induced cardiomyocyte (GSM1305965-7(vs. Three samples of fibroblasts (GSM1359650-2)	Hand2, Nkx2.5, Gata4, Mef2C, and Tbx5 TFs along with SB431542 small molecule were used to convert fibroblasts to Cardiomyocytes	GSE54022	Mouse	Affymetrix Mouse Gene 1.0 ST Array
Qian *et al*.^10^	Two samples of induced cardiomyocyte (GSM1195261-2) vs. Three samples of fibroblasts (GSM1195263-5)	Gata4, Mef2c, and Tbx5 were used to convert fibroblasts to Cardiomyocytes	GSE49192	Mouse	Affymetrix Mouse Gene 1.0 ST Array
Muraoka *et al*.^9^	One samples of induced cardiomyocyte (GSM1370506 (vs. One samples of fibroblasts (GSM1370499)	Gata4, Mef2c, and Tbx5 TFS and miR-133 were used to convert fibroblasts to Cardiomyocytes	GSE56913	Mouse	3D-Gene Mouse Oligo chip 24k

### Direct conversion of fibroblast cells into human mesodermal cells

**Figure 2:**
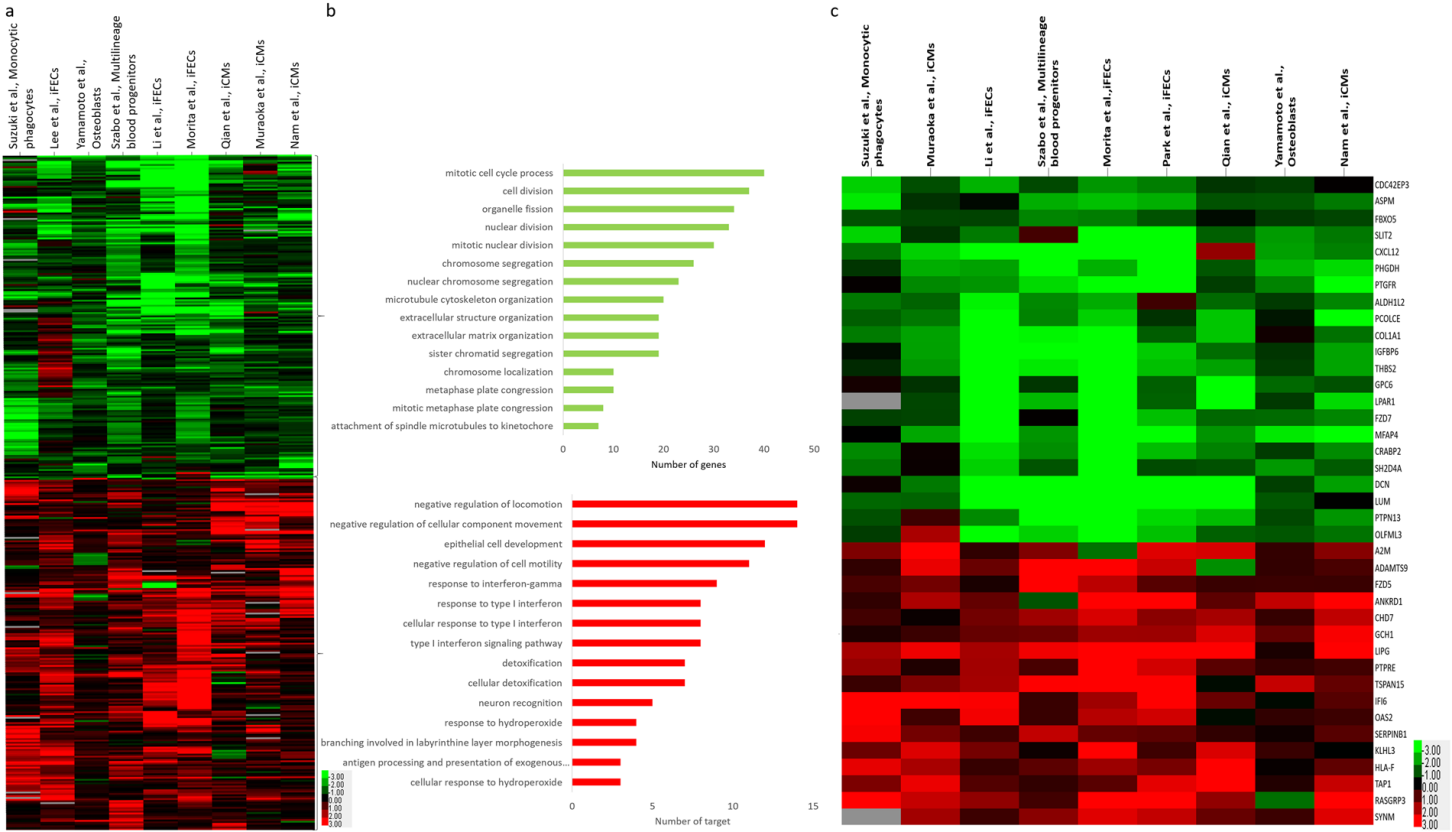
Gene expression clustering of common genes across five different mesoderm-derived cells. (**a**) 379 common up- and down-regulated genes. Different cell types are represented above the graph. (**b**) Ontology results of common up- and down-regulated genes. The most affected terms and number of genes are shown in the Y and X axis respectively. (**c**) The results of expression clustering of 39 genes that were expressed with fold change more than 1.5 in at least 8 out of 9 data sets. In all sections, red indicates up-regulation and green indicates down-regulation of genes.

Because of their abundancy and availability, fibroblasts are the main source of cells used for the direct reprogramming of somatic cells to other cell types. Consequently, expression data sets that have studied the direct conversion of fibroblasts to other somatic cells by different protocols are publicly available. Here, by analyzing and comparing nine expression data sets for the direct conversion of human fibroblast to osteoblasts, MBP, endothelial cells, monocytic phagocytes, and cardiomyocytes, we found that 379 genes had the same expression pattern in at least eight out of nine data sets (Fig. 2a). Ontology analysis of the down-regulated genes in this list showed that the majority of genes were involved in two main categories: the regulation of cell cycle genes and in extracellular matrix organization (Fig. 2b). On the other hand, ontology analysis of the up-regulated genes showed that the negative regulation of cellular component movement and the interferon signaling pathway were the terms with the most significant *p-values* (Fig. 2b). From 379 genes that had the same expression pattern, 22 genes were down-regulated and 17 genes were up-regulated in all converted cells in at least eight out of nine data sets with expression fold change more than 1.5 (Fig. 2c, Supplementary Dataset S1). From the down-regulated gene list, the *CDC42EP3* gene is interesting as it has a role in the remodeling and reorganization of fibroblast cell^29^. Therefore, the down-regulation of this gene indicates the universal conversion of fibroblasts to other cells. Interestingly, in the up-regulated gene list, we found three members of the type I interferon signaling pathway, including the *IFI6*, *OAS2*, and *HLA-F* genes.

**Figure 3:**
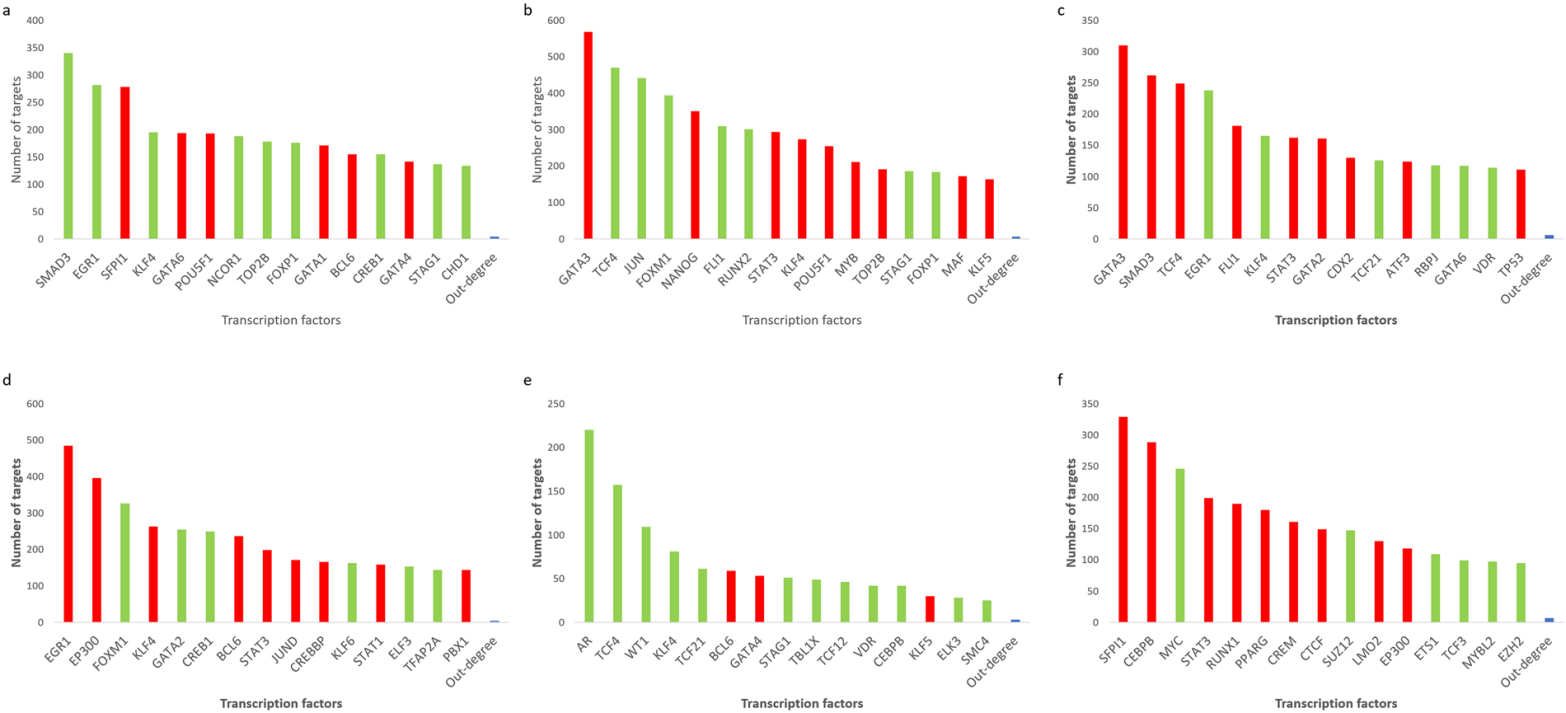
Centrality analysis results and the main TFs which affect the direct reprogramming of six different conversions. (**a**) Fibroblasts to osteoblasts (**b**) Fibroblasts to multilineage blood progenitors (**c**) Fibroblasts to endothelial cells (**d**) Fibroblasts to monocytic phagocyte (**e**) Fibroblasts to induced cardiomyocytes (**f**) Pre-B cells to macrophages. The number of targets is represented in the Y axis and TFs in the X axis.

**Figure 4:**
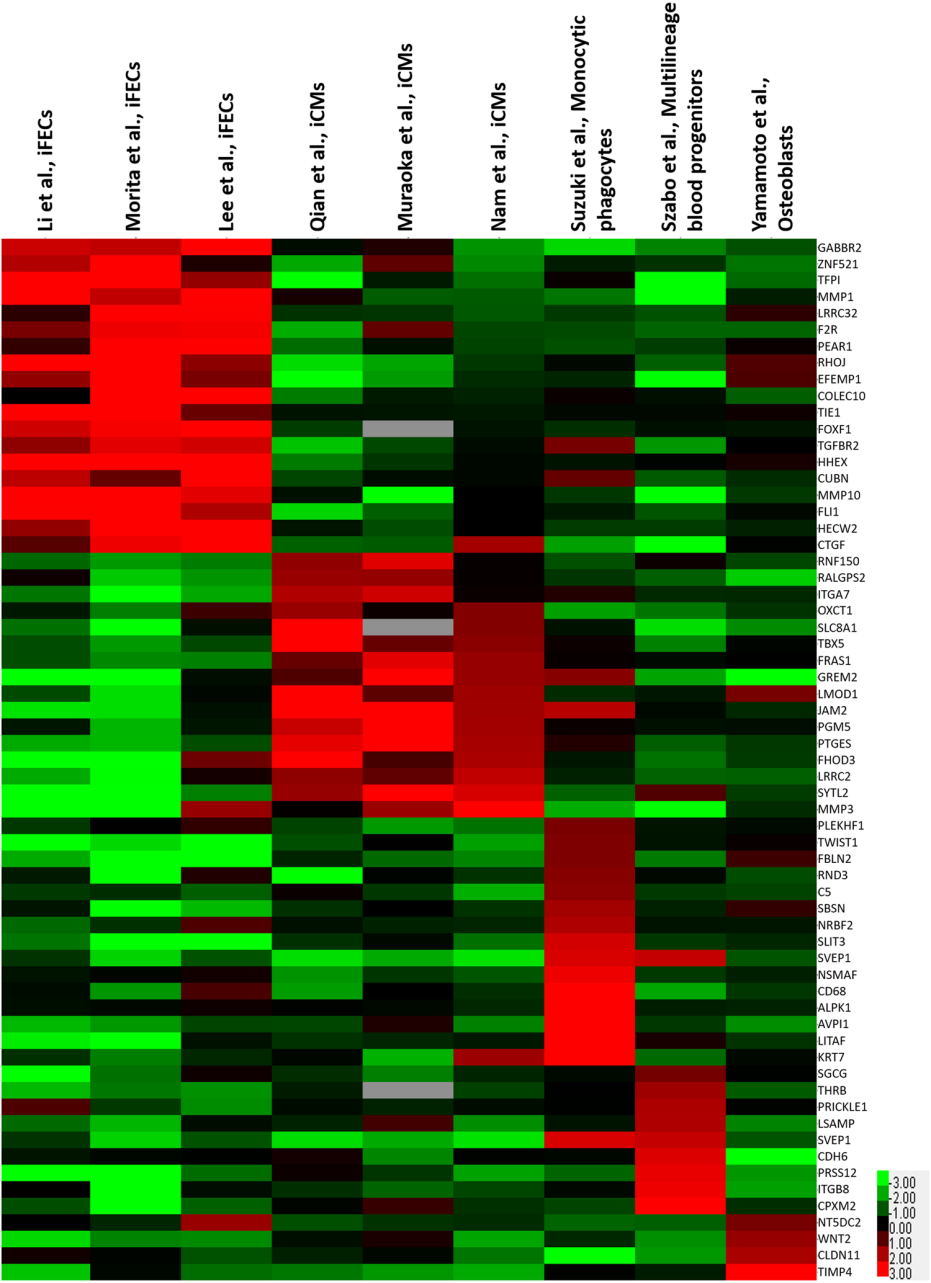
Specific expression pattern for fibroblast-derived cells. The name of each data set is indicated above the graph and gene names are shown in the right side of the graph.

Besides the identification of common genes that were differentially expressed across all converted cells, we considered the direct reprogramming of fibroblasts to different cells on a case-by-case basis and identified the genes that were exclusively expressed in each converted cell. In the direct conversion of fibroblasts to osteoblasts we found 1284 DEGs with *p-value* < 0.05 (Supplementary Dataset S2a). Ontology analysis of DEGs revealed that among the terms with *p-value* < 0.05, there were 45 genes in the ossification term. Furthermore, ontology analysis of up-regulated genes in osteoblasts had good consistency with osteoblast differentiation so that, among the terms that had the most significant *p-values*, there were terms related to response to transforming growth factor (TGF)-beta stimulation. TGF-beta is one of the most fundamental signaling pathways to play a role in bone formation during mammalian development^30, 31^. Transcription factor binding site data analysis showed that 35 differentially expressed-transcription factors (DE-TFs) controlled the expression of DEGs. Based on these 35 DE-TFs, we constructed a GRN for the conversion of fibroblast to osteoblast and found that *SMAD3*, *EGR1*, *SFPI1*, *KLF4* and *GATA6* TFs were the most central TFs that controlled the expression of DEGs in the network (Fig. 3a). Appropriately, the SMAD family of proteins and signaling pathway have significant roles in skeletal development and regeneration^32^. To find a specific gene expression profile for osteoblasts, we compared the 1284 DEGs with eight data sets related to four other cell types, including MBPs, monocytic phagocytes, endothelial cells, and cardiomyocytes, and we found that 53 DEGs expressed differentially in at least seven out of eight data sets, and thus could be considered osteoblast specific genes. Surprisingly, from these 53 genes, only four genes, including *NT5DC2*, *WNT2*, *CLDN11*, and *TIMP4* were up-regulated while the remaining genes were down-regulated in induced osteoblasts (Fig. 4, Supplementary Dataset S3).

For the conversion of fibroblasts to MBPs, we found 1295 DEGs between fibroblasts and MBPs (Supplementary Dataset S2b). Ontology analysis of DEGs and especially down-regulated genes showed that the majority of genes were involved in extracellular organism development and cell movement related process which indicates that during direct reprogramming fibroblast cells undergo significant morphological changes. Incorporating transcription factor-binding site data showed that 44 DE-TFs governed the expression of DEGs in this conversion. Using the data of these 44 DE-TFs and their targets, we constructed the GRN and showed that *GATA3*, *TCF4*, *JUN*, *FOXM1*, and *NANOG* were the most central genes that had roles in the regulation of DEGs (Fig. 3b). From 1295 genes, the expression of 16 genes were specific to MBP, so that these 16 genes expressed with fold change more than 1.5 in at least seven out of eight data sets (Fig. 4, Supplementary Dataset S3). *ITGB8* was an up-regulated gene in the specific gene list, and abnormal expression of this gene has been shown to give rise to severe abnormalities. For example, conditional deletion of *Itgb8* in hematopoietic cells and and dendritic cells of mice induced late-onset autoimmune syndrome characterized by splenomegaly, hepatitis, and colitis with death between 4 and 10 months of age^33^.

In another conversion, we considered the direct conversion of fibroblasts to endothelial cells. For this conversion, there were three independent data sets, each of which were produced separately. The criteria to select DEGs in this conversion was that a DEG should be expressed differentially with the same expression pattern in at least two out of three data sets, and that in the third data set there should not be opposite differential expression. By these criteria, we found 676 DEGs between fibroblast and endothelial cells (Supplementary Dataset S2c). Ontology analysis of the overall list of DEGs showed that the most affected biological pathways that underwent alteration in this conversion were those involved in cell migration, cell motility, cardiovascular system and angiogenesis related terms. The fact that these terms appeared with the lowest *p-value* in our ontology analysis suggests a good consistency with the function and nature of endothelial cells, which are known to have significant roles in the formation of blood vessels and in the development of the circulatory system. Furthermore, we found 43 DE-TFs that had roles in the regulation of the expression of DEGs in this conversion. Among these 43 DE-TFs, *GATA3*, *SMAD3*, *TCF4*, *EGR1*, and *FLI1* were the five top central regulators that had the most targets in this conversion (Fig. 3c). For endothelial cells, we identified 30 genes with exclusive differential expression, from which 19 and 11 genes were up- and down-regulated respectively (Fig. 4, Supplementary Dataset S3). One of the most important genes that was up-regulated was *FLI1*, a TF which has a crucial role in the development of endothelial cells^34^. Therefore, we can consider this gene to be an important marker that could be used to track the progression of the direct conversion of fibroblasts to endothelial cells.

The direct conversion of fibroblasts to monocytic phagocytes composed another conversion of our study. In this conversion, we found 1522 DEGs (Supplementary Dataset S2d), and ontology analysis of this list showed macrophage function related terms to be among the most affected terms. For example, defense response and response to cytokines were two terms that were dramatically altered. Besides macrophage related terms, cell motility related terms also comprised a significant portion of the most altered processes and pathways. We browsed the overall list of DEGs in the database of protein-DNA interaction and found that 39 DE-TFs controlled the expression of DEGs. Among these 37 DE-TFs, *EGR1*, *EP300*, *FOXM1*, *KLF4*, and *GATA2*, were the five central regulators based on centrality analysis of the GRN (Fig. 3d). The expression of 15 up-regulated and 7 down-regulated genes were found to be restricted to monocytic phagocytes, as their expressions changed by fold change more than 1.5 in eight out of nine data sets (Fig. 4, Supplementary Dataset S3). From these specific genes, up-regulation of SLIT3 gene was especially interesting, as it has a role in regulating the cell motility of macrophages^35^.

**Figure 5:**
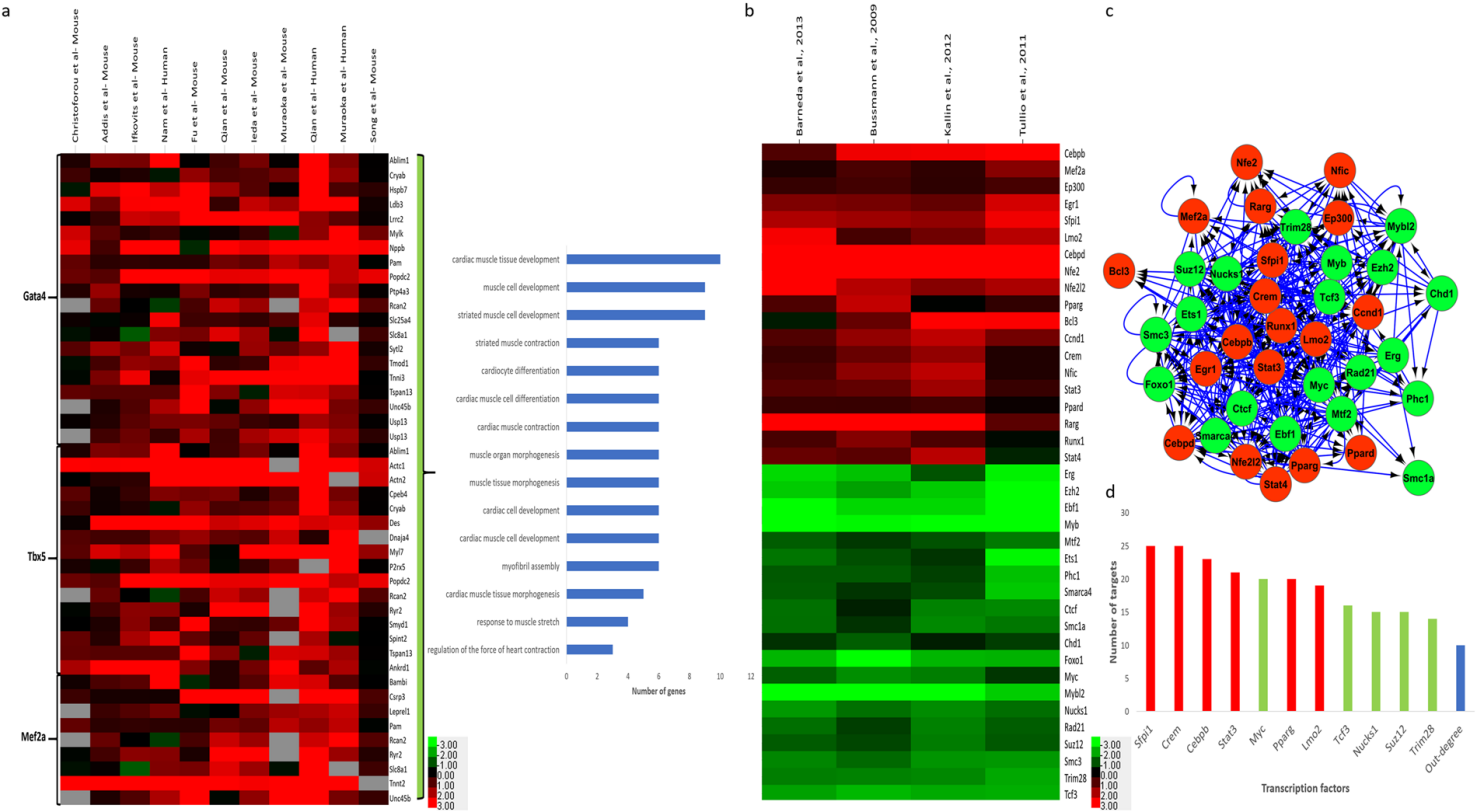
(**a**) Expression correlation of targets for Gata4, Tbx5 and Mef2a between humans and mice for the direct reprogramming of fibroblasts to induced cardiomyocytes. The selected differentially expressed genes in mouse should have expression fold change more than 1.5 in at least four out eight data sets and for humans should have differential expression in at least two out of three data sets. Ontology results of selected genes are represented in the right side of the graph and are ordered based on *p-value*. (**b**) Gene expression clustering of 39 DE-TFs identified during the conversion of Pre-B cells to macrophages (**c**) Core gene regulatory network between DE-TFs. The arrows show the direction of interactions. (**d**) Centrality analysis of DE-TFs core regulatory network. The number of targets are represented in the Y axis and TFs in the X axis.

The last conversion that we analyzed in this section was the direct conversion of fibroblasts to induced cardiomyocytes. We previously analyzed this conversion in detail in a previous paper^28^, however we did not compare them to other mesodermal lineage cell types, which would further enhance the specificity of the results. Therefore, for further clarification on this conversion, first we performed analysis on three data sets, with the same criterion as the conversion of fibroblasts to endothelial cells. Briefly, we found 391 DEGs between fibroblasts and induced cardiomyocytes (Supplementary Dataset S2e). As before, functional analysis of identified DEGs in this conversion showed that the most affected biological terms and pathways were related to muscle structure development, myofibril assembly, and circulatory system development. However, the comparison of induced cardiomyocytes with other mesodermal cells yielded 23 cardiomyocyte- specific genes(Fig. 4, Supplementary Dataset S3). A crucial gene which was up-regulated in the cardiomyocyte-specific gene list was *FHOD3*, which has a fundamental role in the maintenance of contractile structures of heart muscle^36^. Therefore, we suggest that this gene be used as a marker to follow the process of direct conversion of fibroblasts to induced cardiomyocyte. Furthermore, based on the data of transcription factor-binding sites, 17 DE-TFs controlled the expression of DEGs. Centrality analysis of the GRN showed that *AR*, *TCF4*, *WT1*, *KLF4* and *TCF21* were the five central TFs (Fig. 3e). In order to further expand the comprehensiveness of these results and of our previous results^28^, besides analyzing human samples, we also analyzed mouse samples. To this end, we analyzed the data of the direct reprogramming of mouse fibroblasts to induced cardiomyocytes and we revealed interesting results. For example, we found more regulatory interactions for the GATA4 TF (Fig. 5a) and identified important TFs that were not found in human samples, such as *TBX5*, and *MEF2A*. These TFs have significant roles in the generation of cardiomyocytes and were up-regulated during cardiomyocyte induction (Fig. 5a). There was a good correlation between target genes of these TFs in human and mouse sample data sets (Fig. 5a). The existence of such correlation between the two organisms indicates that the obtained results from the analysis of one organism could potentially be applied for another one.

Altogether in this part, we analyzed the direct conversion of human fibroblasts to five different cell types, and for each conversion, we identified DEGs, the most affected pathways, and the TFs that control the expression of DEGs. Furthermore, we constructed GRNs based on TF binding site and expression data and performed centrality analysis to find the most central TFs in each conversion. Finally, we highlighted specific expression profiles for each cell. In the remaining part of the paper, we analyze the direct conversion of various mesoderm layer cell types to other mesoderm layer cell types for both humans and mice.

### Generation of mesodermal cells from non-fibroblastic cells

In this section, we present our results on the analysis of the remaining conversions for both human and mouse samples, including amnion cells to induced chondrocyte (Supplementary Table S1, Figs S1a, S2a, Supplementary Dataset S2f), endothelial progenitors to smooth muscle cells (Supplementary Table S1, Figs S1b, S2b, Supplementary Dataset S2g), white adipocytes to brown adipocytes (Supplementary Table S1, Figs S1c, S2c, Supplementary Dataset S2h), T cells to natural killer-like cells (Supplementary Table S1, Figs S1d, S2d, Supplementary Dataset S2i), pre-B cells to macrophages (Supplementary Dataset S2j), myoblasts to brown fat cells (Supplementary Table S1, Figs S1e, S2e, Supplementary Dataset S2k), granolosa to sertoli cells (Supplementary Table S1, Figs S1f, S2f, Supplementary Dataset S2l), and fibroblasts to embryonic sertoli-like cells (Supplementary Table S1, Figs S1g, S2g, Supplementary Dataset S2m). For all conversions, we identified DEGs, the DE-TFs that control DEGs expression, the most central TFs, and finally the most affected biological pathways. We largely concentrated on the generation of mouse macrophages from Pre-B cells.

In order to analyze the direct conversion of pre-B cells to macrophages in mice, we used four microarray data sets which were produced independently^15–18^. To be considered common DEGs, genes should be expressed differentially in at least three out of four data sets and have the same expression pattern. By this criterion, we found 522 genes for which 250 genes were down-regulated and 272 genes were up-regulated (Supplementary Dataset S2j). Ontology analysis of up-regulated genes in this conversion clearly showed that macrophage related terms were altered significantly. On the other hand, interestingly, ontology analysis of down-regulated genes indicated that the majority of affected processes were related to cell cycle pathways. As pre-B cells show more proliferation activity than macrophages, we conclude that the occurrence of cell cycle related terms among terms that were down-regulated shows good consistency with the process by which a more potent cell converts to a differentiated cell. Furthermore, to gain more insight on the regulation of the gene expression program, we sought to identify TFs that controlled the expression of DEGs. To this end, we found that 39 DE-TFs took part in controlling the expression of DEGs. These 39 TFs expressed differentially in at least three out of four data set with identical expression patterns and with a fold change more than 1.5 (Fig. 5b). A GRN was constructed based on these DE-TFs that also have roles in gene expression regulation of DEGs and was subjected to centrality analysis, and *Sfpi1*, *Cebpb*, *Myc*, *Stat3* and *Runx1* were found to be the most central genes that contained the most number of targets (Fig. 3f). Recently, it was shown that *Cebpb* along with *Bach2* gene may form a regulatory circuit which affects multipotent blood progenitor cell fate^37^. Finally, to gain a better insight into regulatory interactions between DE-TFs, we constructed a core regulatory network which governs the expression of 39 DE-TFs (Fig. 5c). Interestingly, in this network *Sfpi1* significantly regulated the other TFs and ranked as the most central TF (Fig. 5d). Following *Sfpi1*, *Crem* ranked as the second-most central TF. Unexpectedly, *Crem* was among the top five central TFs that controlled the expression of the overall list of DEGs (Fig. 3f).

Taken together, in this study, we analyzed the direct conversion of human and mouse mesoderm cell lineages to each other by dissecting the gene expression profile of cells. In the first part, we investigated the direct conversion of human fibroblasts to five different cell types, and for each conversion, we identified an overall list of DEGs, a list of genes that expressed solely in one cell type that we believe could be used as markers to follow process of the direct conversion, we found the DE-TFs that control the expression of DEGs and ranked them based on centrality analysis of the GRN, and finally we revealed the most affected biological processes for each conversion. In the second part, we identified a list of common genes that were down- and up- regulated across all the converted cells. Common down regulated genes were mainly involved in cell cycle regulation and extracellular matrix organization. Meanwhile, up-regulated genes had roles in the interferon signaling pathway and the negative regulation of cell movement. We suggest that the function of identified common genes is to suppress the fibroblast-specific gene expression program and to direct cells toward their specific fate. For each conversion, we performed the same set of analyses as fibroblast-derived cells for humans, and found the specific genes for each unique conversion. In addition to considering human fibroblast-derived cells, we also studied the direct conversion of other cells in humans and mice for which, the cell of origin was not the same (Table 1).

## Discussion

The direct reprogramming of somatic cell to other somatic cell types is a promising approach in the field of regenerative medicine. In this study, we used 27 freely available human and mouse expression data sets to evaluate the direct reprogramming of the mesoderm layer cells to each other. We represented our results in two separate sections. In the first part, we presented the direct reprogramming of human fibroblasts to osteoblasts, multilineage blood progenitors (MBPs), monocytic phagocytes, endothelial cells, and cardiomyocytes, and in the second section, we analyzed various somatic cell conversions in the mesoderm layer from non-fibroblastic cells, with specific attention to the conversion of mouse pre-B cells to macrophages. For the direct conversion of mesodermal cells from fibroblasts, we identified a common profile of genes which have the same expression pattern across all cells. In addition, for each cell type, we identified distinct profiles of genes that played roles in cell-specific functions. Afterwards, we recognized transcription factors (TFs) that governed the transition of cells, constructed gene regulatory networks (GRNs) for each transition, and ranked TFs based on their importance. Finally, we identified the biological processes that were significantly affected by each transition. Here, we will discuss our results in more detail.

During the conversion of human fibroblasts to five different fibroblast-derived mesodermal cell types, there were groups of genes that were significantly down-regulated in all converted cells. The majority of those genes have roles in two main biological processes, which were the regulation of cell cycle genes and extracellular matrix organization. In addition, we found a group of genes that was up-regulated across all cells. These genes mainly took part in the negative regulation of cellular component movement and in interferon signaling pathway. The combination of down- and up-regulated gene ontology results overlapped and led to the same interpretation. For example, the interferon signaling pathway is one of the most significant terms in up-regulated genes ontology and has suppressive effects on cell proliferation^38^. This has a meaningful overlap with the ontology results that showed that cell cycle genes were some of the most down-regulated genes. Therefore, the combinatorial effect of these two affected pathways indicates that during the direct conversion of fibroblasts to different mesodermal layer cells, there is a decrease in cell proliferation activity, or that the vast number of genes dynamically deal with the quiescence of the fibroblast gene expression program. In the common down-regulated genes, THBS2 is a gene known to affect fibroblast motility. Yang and colleagues previously revealed that Thbs2 null mice showed a deficit in binding to the extracellular matrix and had attenuated cell spreading^39^. Besides THBS2, other less known genes that affect extracellular matrix organization could be considered markers of fibroblasts. For instance, the *SLIT2* gene was originally known as a highly conserved gene with a role in axonal guidance and neural migration^40, 41^. Recently however, Pilling and colleagues showed that human lung fibroblasts produced *SLIT2* ^42^. *SLIT2* might have a role in other aspects of fibroblasts, as we showed that its down-regulation in fibroblasts direct their conversion into five different cells. Therefore, we can conclude that the most down regulated gene list, including THBS2 and SLIT2, could have a role in initiating the direct conversion process and should be precisely regulated in order to achieve a high quality of converted cells. In the common up-regulated genes, we identified *ILI6*, *OAS2* and *HLA-F* genes as members of the type I interferon signaling pathway. In the ontology results of up-regulated genes, we revealed that interferon signaling pathway is a term that is affected ubiquitously in all cells. The type I interferon signaling pathway has contradictory roles in cell cycle regulation. For example, despite previous reports which showed suppressive effects of interferon on cell proliferation^43, 44^, there are other reports which confirm the role of type I interferons in stimulating the proliferation of hematopoietic stem cells (HSC)^45, 46^ and endothelial cells^47^. Taken together, monitoring the expression of the common genes that have the same expression pattern in all converted cells could be a good strategy to distinguish the initiation of the direct reprogramming process from other steps, and further studies into their molecular mechanisms could be beneficial for our understanding.

In the next part, we found lists of specific genes which have roles in the functional properties in each of the five unique cells that were directly generated from fibroblasts. For instance, in the direct conversion of fibroblasts to osteoblasts, we found four genes to be specifically up-regulated only in osteoblasts. *TIMP4* was one of the up-regulated genes in that conversion and has a significant role in osteogenesis. In a fundamental study, Fleinken and colleagues recognized the expression of the TIMP family of proteins at day E13.5 of mouse embryo in tissues undergoing osteogenesis, such as the mandible, ribs, and calvaria^48^. Besides this, the TIMP family is characterized by their metalloproteinase inhibitory effect^49^, a feature that is in a good agreement with the ontology results we obtained, in that one of the most significantly altered biological process was the down-regulation of cell movement related terms. For endothelial cells, we showed that the expression of the *TIE1* gene is increased significantly. This gene has a crucial role in the embryonic development of the endothelial cell^50^. By conjugating this gene with green fluorescence protein (GFP), Iljin and colleagues established a model for embryonic vascular development and created an efficient strategy to isolate endothelial cells^51^. Therefore, as we identified an acute up-regulation of this gene, we suggest that *TIE1* can be considered a good marker to follow endothelial cell maturation, including the specificity of the reprogramming.

The identification of master regulators of each conversion was another section of our analysis, which we performed by analyzing gene regulatory networks (GRNs). For example, in the conversion of fibroblasts to the different endothelial cells, we identified *SMAD3* as the most central gene in the concept of the GRN. Previous studies have shown the involvement of *SMAD3* gene in the establishment of angiogenesis^52^. Lebrin and colleagues showed that during the second phase of angiogenesis, the *SMAD3* gene becomes activated and stabilizes the integrity of endothelial cells in blood vessels^52^. For the conversion of fibroblasts to induced cardiomyocytes, *GATA4* appears to play an important role. This TF has significant roles in the generation of cardiomyocytes^53, 54^, a role for which we previously highlighted in detail during the direct reprogramming of mouse and human fibroblasts to cardiomyocytes^28^. In addition to the TFs that we identified in our results, TFs that control cardiogenesis in mice could be applied to the human samples. For example, it is known that *Tbx5* and *Mef2a* are three master regulators that control the development of cardiomyocyte in mouse^55, 56^. We also showed that *TBX5*and *MEF2A* had significant expression changes in human samples. However, as these TFs did not have transcription factor binding site data in humans, we could not identify them as main regulators in this study^28^. These three TFs were significantly up-regulated in human induced cardiomyocytes and their targets had good expression correlation with mouse data, so that we detected extensive over-expression of their targets in both species (Fig. 5a).

In the current study, we proposed a new comprehensive approach through the analysis of the expression profile of cells in order to dissect the direct conversion of somatic cells to each other in mesoderm layer cells. This approach could be applied to any kind of cell conversion^28^. This study was composed of two parts. In one part, we analyzed the conversion of five human mesoderm cell types from a common fibroblastic origin. For this step, we found a common list of down-regulated and up-regulated genes across all converted cells. As these genes have the same expression pattern in all cells and are involved in inhibiting fibroblast-specific features, we can assume that they may act as general markers for the initiation stage of direct reprogramming. Moreover, we found an expression profile that was limited to each cell individually. Ontology analyses of the specific genes showed that these genes were mainly involved in the functional properties of the converted cells. In this regard, we believe that these specific genes can be considered potential markers to follow the direct reprogramming process, and may be useful to screen and distinguish the initiation and maturation steps of each conversion. Furthermore, in the next steps, we identified the TFs that acted as the general regulators of the gene expression program and that affect the process of the direct reprogramming, and ranked them based on their importance by constructing and analyzing their GRN. Our approach has the advantage of identifying down-regulated genes that have roles in the progression of the direct reprogramming process (especially TFs), a feature that is absent in other current powerful strategies^25^. The presence of down-regulated TFs seems to be important for the progression of the direct conversion process, as our ontology results revealed there was a good correlation with related down- and up-regulated genes. Despite our advantage of being able to identify down-regulated TFs, our approach has the same disadvantage as other protocols in that this approach also requires the existence of previously available data. For example, we identified that the expression of Mef2c is changed significantly during conversion of fibroblasts to induced cardiomyocytes. Although, Mef2c has a significant role in cardiogenesis^57^, there is no TF-binding site for this TF in human nor in mouse and therefore, we could not find this TF as master regulators of DEGs. Taken together, to the best of our knowledge, we have for the first time applied a comprehensive approach to analyze the direct reprogramming of mesodermal cell types to each other using the application of freely available transcriptome data sets. Our approach is an efficient and useful approach to identify important common or specific DEGs to screen for the initiation and maturation of the direct conversion process. Furthermore, the use of our identified DE-TFs, especially the ones central for direct reprogramming, will be potentially useful to increase the efficiency and functionality of generated cells. Finally, such analyses shed light on the molecular mechanisms that underlie the direct reprogramming process and brings about new insight into this area of study.

## Materials and Methods

### Expression data sets availability and analyses

As the first step, 27 freely available human and mouse expression data sets for the direct conversion of mesoderm layer cells to each other were downloaded from the Gene Expression Omnibus (GEO) NCBI database^3–11, 13, 15–19, 21–23, 58–63^ (Table 1). For each conversion, the normalized data was loaded into the Flexarray software and following the experimental design, the fold change algorithm was applied to the data^64^. The fold change algorithm identifies the fold change of expression differences between origin and target cells. Then, the *p-value* parameter was calculated for all genes in each data set and *p-value* < 0.05 was selected as a threshold to identify the differentially expressed genes (DEGs). For those conversions that contained more than one data set, including the direct conversion of human fibroblasts to induced cardiomyocytes and endothelial cells, and the direct conversion of mouse Pre-B cells to macrophages, we considered a gene as a DEG if that gene expressed differentially with the same expression pattern (either up- or down-regulated) in at least two out of three data sets for endothelial cells and induced cardiomyocytes, and in at least three out of four data sets for Pre-B cells to macrophages.

For the conversion of human fibroblasts to osteoblasts, mutlilineage blood progenitors (MBP), endothelial cells, monocytic phagocytes, and induced cardiomyocytes, our criterion for identifying common DEGs was that those genes should be expressed differentially with the same expression pattern in at least eight out of nine independent data sets. Furthermore, we indicated an expression profile that was specific for each converted cell. The specific profile of each gene was composed of a group of genes that not only expressed differentially in target cells, but also had the opposite expression pattern to other converted cells. However, to reduce the occurrence of false negative results, we established a fine-tuning criterion in which each cell-specific gene could be expressed with identical patterns in a maximum two other data sets. As an example, for induced cardiomyocytes, the cell-specific gene should have opposite expression patterns in at least seven out of nine other data sets, but might have the identical expression patterns in a maximum of two out of nine data sets.

### Construction of gene regulatory networks (GRNs) using transcription factor-binding site data derived from high throughput experiments

The data of transcription factors (TF)-binding site were obtained from the ChIP Enrichment analysis (ChEA) database^65, 66^. ChEA contains TF-DNA interactions data obtained from ChIP-chip, ChIP-seq, ChIP-PET, and DamID. In the most recent version, this database contained 645 independent high throughput TF-binding site data sets for a wide range of TFs. We perused the DEGs list in this database and retrieved TFs that controlled the expression of DEGs, and kept TFs that contained *p-value* < 0.05. In the case of TFs, we set another level of filtering to identify differentially expressed TFs (DE-TFs) by setting expression fold change to 1.5 and selecting those as DE-TFs. Using TF-binding site data of the DE-TFs, DE-TF expression, and DEGs, the GRNs were constructed for each conversion in the Cytoscape software^67^.

### Highlighting the most central genes and the most affected processes

The constructed GRNs were subjected to two different analyses to find the most central genes and the most affected biological processes in the concept of the GRN for each conversion. The most central genes were identified using the CentiScaPe plug-in of the Cytoscape software on the constructed GRN. CentiScaPe considers the direct interaction of each TF with its neighbor as a degree parameter^68^. Out-degree was applied on the constructed directed GRN for each conversion. Out-degree shows which DE-TFs have the most number of targets and these DE-TFs are considered as the most central or hub TFs. In addition, we identified the most affected biological process during each conversion using ontology analysis. To this end, the ClueGO and CluePedia plug-ins of the Cytoscape software were used and in the advanced statistical option of the tools, two-sided hypergeometric test was selected to calculate the importance of each term and Bonferroni step-down was used for *p-value* correction^69, 70^. The terms with *p-value* < 0.05 were considered as meaningful terms.

### Clustering of differentially expressed genes

In order to evaluate the degree of similarity between human fibroblast-derived cells and to perform co-expression analysis of the DEGs, the Gene Cluster^71^ and Java TreeView^72^ softwares were used to cluster the converted cells and DEGs and to visualize the results, respectively. We used hierarchical clustering, which is a strong method for analyzing high throughput expression data. Similarity metrics were calculated for both genes and arrays. To measure the similarity of both arrays and genes, correlation (un-centered) was used.

## Electronic supplementary material

Supplementary files

Dataset 1

Dataset 2

Dataset 3
