# Supplementary material for: Comprehensive transcriptome mining of the direct conversion of mesodermal cells
Source: Sci Rep. 2017 Sep 5;7:10427. doi: 10.1038/s41598-017-10903-z (PMC5585404; doi:10.1038/s41598-017-10903-z)

Comprehensive transcriptome mining of the direct conversion of mesodermal cells

Bijan Akbari, Ping Wee, Moein Yaqubi, Abdulshakour Mohammadnia

Supplementary Tables

1. Supplementary Table S1: the list of differentially expressed transcription factors in fibroblastic and non-fibroblastic mesodermal cells direct conversion.

Supplementary Datasets legends:

1. **Supplementary Dataset S1: the list of common DEGs for conversion of fibroblasts to human mesodermal cells.**

2. **Supplementary Dataset S2a-m: the list of differentially expressed genes for conversion.**

Supplementary Dataset S3: the list of specific DEGs for each fibroblast-derived cells.

Supplementary Figures:

1. **Supplementary Figure S1a-g: the gene ontology analysis of amnion cells to induced chondrocyte (a), endothelial progenitors to smooth muscle cells (b), white adipocyte to brown adipocyte (c), T cells to natural killer-like cells (d), myoblasts to brown fat cells (e), granolosa to sertoli cells (f), and fibroblasts to embryonic sertoli-like cells (g). For T cells to natural killer-like cells (d) and fibroblasts to embryonic sertoli-like cells (g) ChEA 2015 was used.**

2. Supplementary Figure S2a-g: the centrality analysis of the gene regulatory networks for amnion cells to induced chondrocyte (a), endothelial progenitors to smooth muscle cells (b), white adipocyte to brown adipocyte (c), T cells to natural killer-like cells (d), myoblasts to brown fat cells (e), granolosa to sertoli cells (f), and fibroblasts to embryonic sertoli-like cells (g).

1. Supplementary Table S1: the list of differentially expressed transcription factors in non-fibroblastic mesodermal cells direct conversion.

Conversion type	Organism	Nr. of TFs	Up-regulated TFs	Down-regulated TFs
Endothelial progenitor to smooth muscle cell	Human	43	FOXA1, NFE2L2, STAT1, VDR, KDM6A, CEBPB, CUX1, BCL6, MITF, EGR1, AHR, GATA6, KLF5, TFAP2A, MAF, ATF3, GBX2	FOXM1, SMC4, RAC3, NOTCH1, TTF2, FLI1, TRIM28, GATA3, MYC, FUS, STAG1, TCF4, HIF1A, ERG, AUTS2, DNAJC2, NFYB, TDRD3, TBL1X, DACH1, E2F1, DROSHA, ZMYND8, IRF1, TAF15, CTCF
amnion cells to induced chondrocyte	Human	92	E2F4, NFYA, PADI4, TOP2B, CHD1, BACH1, FOXP2, WDR5, YY1, FOXM1, NR1H3, MAF, FUS, SOX2, ELF5, TAL1, ESR2, GATA1, IRF8, PAX3, GATA4, PRDM14, UBTF, TP63, FOXH1, NR3C1, HNF4A, DNAJC2, FOXA2, CBX2, EOMES, SMC4, TCF3, PHF8, EZH2, TAF15, TRIM28, E2F1, IKZF1, KLF5, CDX2, BCOR, VDR, MYCN, RUNX2, SALL4, SOX11, POU2F1, MYB, RAC3, ATF3, ETV1, GATA3, POU3F2, PRKCQ, TCF21, FOXA1	BCL6, AR, FLI1, TFAP2A, GATA2, WT1, GBX2, STAT1, HIF1A, ERCC6, MYC, GATA6, IRF1, ELK3, MITF, TP53, TFAP2C, ELK1, CLOCK, FOXO3, ELF3, STAT3, POU5F1, PIAS1, STAT6, CREB1, RUNX1, ARNT, STAG1, CTBP2, CUX1, NCOR1, HOXB7, RELA, NFE2L2
Fibroblasts to osteoblasts	Human	35	RAC3, SPI1, FUS, PADI4, GATA6, KLF6, FOXO3, DROSHA, GBX2, PRDM14, GATA1, NCOR2, BCL6, GATA4, POU5F1	SMC4, ETV1, TOP2B, NR3C1, CREB1, IRF1, SMAD3, NFE2L2, STAG1, EGR1, CHD1, DDX5, KLF4, TFAP2C, TFAP2A, CLOCK, NCOR1, TCF12, FOXP1, IKZF1
Fibroblasts to multilineage blood progenitors	Human	44	NANOG, KLF4, AHR, KDM6A, DDX5, MAF, POU3F2, IRF1, BCOR, POU2F1, TOP2B, TP63, CTNNB1, HTT, IRF8, GATA3, NFE2L2, ZNF217, TP53, KLF5, MYB, STAT3, ERCC6, ELF3, PAX3, SALL4, TFAP2C, POU5F1	TCF4, ELK3, KLF6, JUN, STAG1, FOXP1, SMC4, TBL1X, ETV1, RUNX2, FOXM1, FLI1, NR3C1, HIF1A, AUTS2, HOXC9
Fibroblasts to endothelial cells	Human	43	ATF3, CBX2, CDX2, CTNNB1, ELF1, ERG, ESR2, FLI1, GATA2, GATA3, GBX2, HOXB7, HTT, IRF1, JUND, KLF6, NOTCH1, POU2F1, PRKCQ, RAC3, SMAD3, STAT3, TAL1, TCF3, TCF4, TP53, ZMYND8	AUTS2, CEBPB, EGR1, FOXO3, GATA6, KLF4, KLF5, MAF, NR1H3, NR3C1, PBX1, RBPJ, SMC4, SOX11, TCF21, VDR
Fibroblasts to monocytic phagocytes	Human	39	JARID1A, PBX1, PIAS1, DDX5, TBL1X, ELF1, NFE2L2, CREBBP, EP300, KLF4, BCL6, IRF1, JUND,	FOXM1, SMC4, HIF1A, PRKCQ, ETS1, ELF3, YY1, TAF15, ZNF652, GATA2, RAC3, CHD1,

			NR1H3, CEBPB, STAT3, STAT6, HOXB7, ZNFN1A1, STAT1, EGR1	STAG1, SOX11, KLF6, CREB1, DCP1A, TFAP2A
Fibroblasts to induced cardiomyocyte	Human	17	BCL6, GATA4, KLF5, WT1	AR, CEBPB, ELK3, IRF1, KLF4, SMC4, STAG1, TBL1X, TCF12, TCF21, TCF4, VDR, ZNF263
White adipocyte to brown adipocyte	Mouse	53	Klf1, Ppara, Rarb, Taf7l, Etv2, Sox11, Eed, Elk4, Pcgf2, Sall4, Tbx20, Sox6	Nfic, Jarid2, Sox17, Ash2l, Rxra, Gata1, Tead4, Trim28, Stat3, Gata2, Mef2a, Pax6, Tal1, Ldb1, E2f1, Tfap2c, Phc1, Gata3, Smad1, Sin3b, Spi1, Sox9, Klf2, Trp63, Olig2, Meis1, Tcf3, Isl1, Wt1, Fli1, Irf8, Egr1, Cebpd, Esrrb, Rnf2, Nanog, Nfe2, Dmrt1, Zic3, Esr1, Irf4
T cells to natural killer-like cells	Mouse	15	Egr1	1 Mtf2, Tcf7, Ets2, Bcl11b, Trim28, Ldb1, Ets1, Sox9, Ccnd1, Rcor1, Gata3, Sin3a, Runx1, Rxra
myoblasts to brown fat cells	Mouse	32	Olig2, Meis1, Sox9, Nfe2, Mnx1, Tal1, Sry, Gata1, Klf4, Isl1, Myb, Rarb, Nkx2-5, Smad1, Sox11, Hoxb4, Mef2a	Stat3, Taf7l, Eomes, Dmrt1, Ccnd1, Pparg, Nanog, Sox3, Cebpb, Fli1, Cebpd, Bcl3, Erg, Hoxa2, Sox2
granolosa to sertoli cells	Mouse	36	Nfic, Asxl1, Ncor2, Zic3, Rara, Stat3, Sox6, Mef2a, Thra, Foxo3, Bcl3, Sin3b, Wt1, Cebpd, Gata1, Dmrt1, Sox9, Nr0b1	Foxo1, Myc, Pparg, Runx2, Foxp2, Ets2, Egr1, Foxp1, Smad1, Erg, Cebpa, Sox11, Cebpb, Tet1, Ets1, Rarg, Mybl2, E2f1, Fli1
fibroblasts to embryonic sertoli-like cells	Mouse	14	Sox6, Tfcp2l1	Fli1, Sox11, Prdm16, Bcl11b, Ebf1, Irf8, Cebpa, Zic3, Lmo2, Foxp2, Mybl2, Nfe2, Tal1
Fibroblasts to induced cardiomyocytes	Mouse	24	Gata4, Eomes, Klf5, Mef2a, Smad1, Tbx5, Tead4, Trp63, Wt1	Cebpa, Cebpd, E2f1, Ezh2, Foxp2, Irf8, Lmo2, Lyl1, Meis1, Mybl2, Myc, Nucks1, Sox9, Spi1, TRP53

1. Supplementary Figure S1a-g

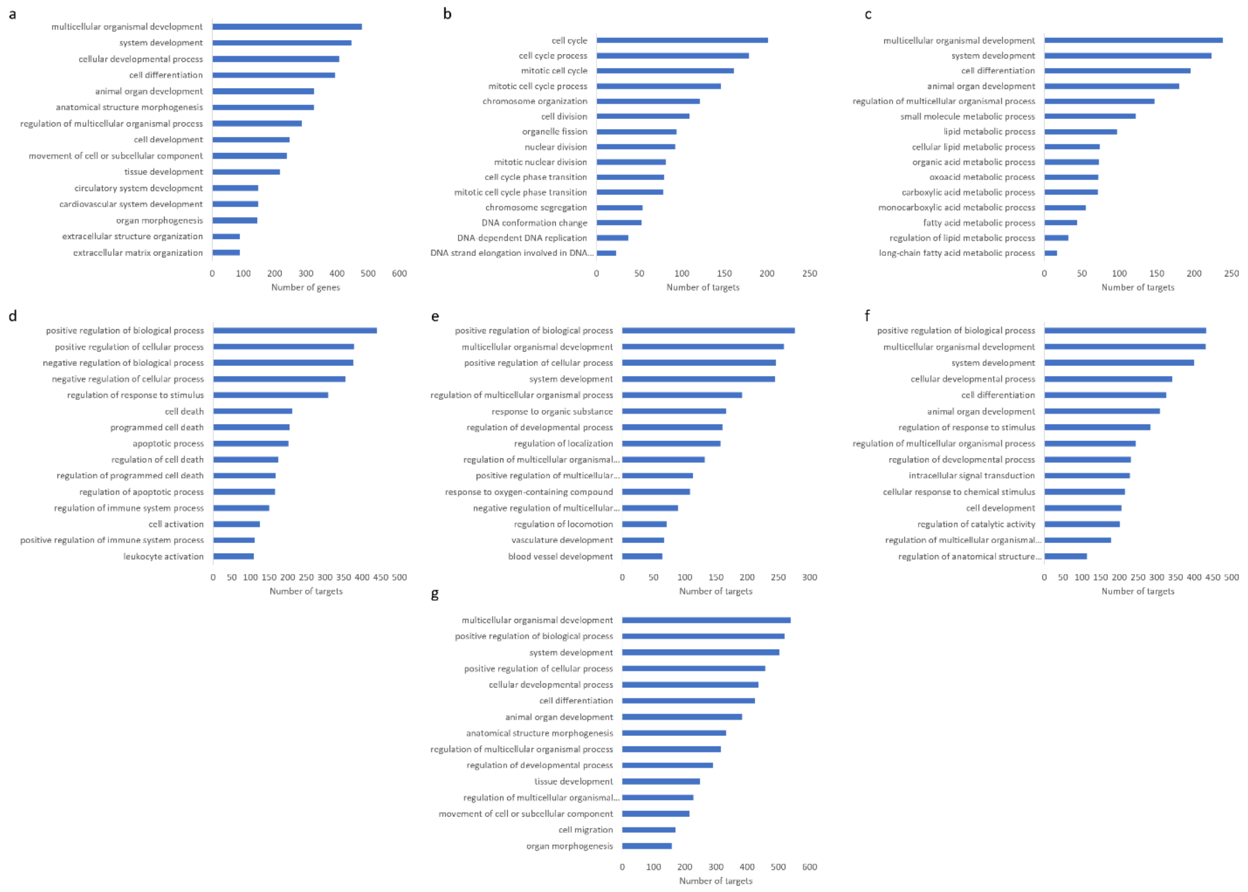

2. Supplementary Figure S2a-g

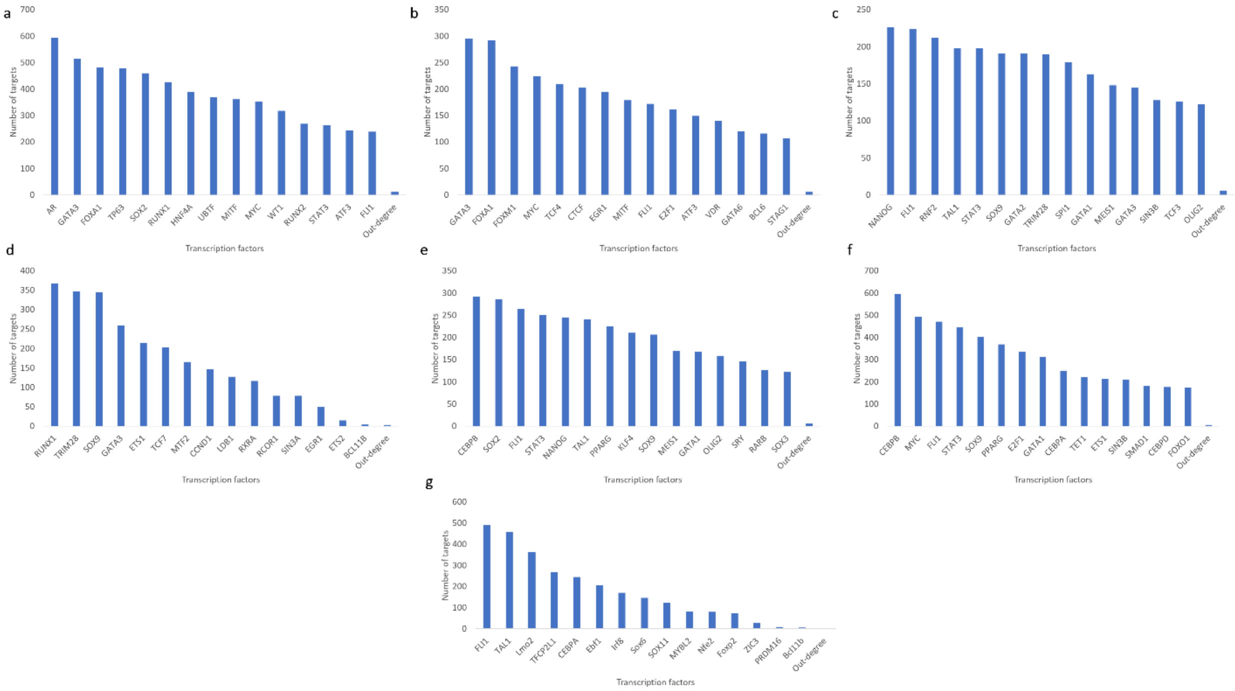

Supplement: Supplementary file 1 — Supplementary files [file 41598_2017_10903_MOESM1_ESM.pdf]
